# The HIV epidemic in eastern Europe and central Asia in difficult times: a story of resilience and change

**DOI:** 10.1002/jia2.26325

**Published:** 2024-07-17

**Authors:** Miłosz Parczewski, Deniz Gökengin

**Affiliations:** ^1^ Department of Tropical, Infectious Diseases and Immunodeficiency Pomeranian Medical University in Szczecin Szczecin Poland; ^2^ Department of Infectious Diseases and Clinical Microbiology, Medical School Ege University Izmir Turkiye; ^3^ Ege University HIV AIDS Research and Practice Center Izmir Turkiye

The eastern European and central Asian (EECA) region is facing the fastest‐growing HIV epidemic in the world with 160,000 (130,000–180,000) people newly acquiring HIV in 2021, an overall 48% increase in the number of new acquisitions, and a 32% rise in AIDS‐related deaths over the past 10 years [1]. The World Health Organization East European region is estimated to accommodate 1.4 million people living with HIV (PWH), with the majority of new transmissions reported to occur locally and to be unrelated to cross‐country migrations [2, 3]. Sadly, according to UNAIDS, in 2022 only 62% of people living in the EECA region were aware of their HIV status and 51% of PWH received HIV treatment, which resulted in an overall viral suppression rate of 48% [1]. Common (>50%) late diagnoses (with either AIDS‐defining condition or CD4^+^ T cell count <350 cells/µl at presentation) [4] further add to the epidemiological issues and complexity of client management [5]. Although the key acquisition risk in the region has changed from unsafe injection practices to heterosexual transmission, access to opioid agonist therapy (OAT) and needle and syringe programmes is often still limited (Kazakhstan) or unavailable (Uzbekistan, Turkmenistan), which results in OAT regional coverage as low as 4%, far below the UNAIDS target of 50%.

Furthermore, pre‐exposure prophylaxis (PrEP) and combination prevention services are of limited availability or not formally implemented across multiple countries in the region usually due to financial barriers to access, such as lack of state funding for medicines, limited technical capacity or cost of service delivery [6]. This results in a substantially larger HIV PrEP gap (the proportion of populations at risk of acquiring HIV “very likely” to use PrEP if accessible, compared with the proportion currently using PrEP) across countries located in the EECA region (up to 45%) compared to the overall median of 17.4% estimate for the European Union [7]. Moreover, there is a high level of stigma across the countries, especially against gay men and other men who have sex with men.

The growing epidemic and the suboptimal access to prevention, testing and treatment are the main reasons that challenge the 2030 targets.

The humanitarian crisis related to the Russian invasion and war in Ukraine has challenged the situation even further hindering testing and treatment efforts, forcing both internal displacement (estimated 3.7 million) and external migration of locals as refugees (estimated 6.4 million, as of March 2024) [8]. On top of massive casualties, civilian and healthcare infrastructures were destroyed, forcing refugees to seek safety, protection and assistance, including continued access to medical care. Ukraine is home to ∼260,000 PWH with >130,000 on antiretroviral treatment (ART). Although significant progress was made in the response to the HIV epidemic in Ukraine before the outbreak of war resulting in a 47% reduction in HIV incidence and an 81% reduction in AIDS mortality, the war has significantly affected the HIV and tuberculosis (TB) programmes, with internal displacement resulting in diminished service capacity, difficulty in ART provision and reduction in prevention services [9]. Despite notable hardship, Ukraine managed to maintain full access to ART, increased OAT rollout by 38% (reaching >27,000 people) and doubled the number of people on PrEP, which is now available free of charge [10].

A large proportion of the studies published in this supplement focus on Ukraine and the responses given during the war, providing good examples of resilience and strength and reflecting personal experiences in the setting of mass trauma. Mass traumas are especially important from the perspective of PWH, as these may bring out stress and exacerbate mental health issues. Owczarzak et al. analyse the bioecological model of mass trauma in the context of the Russian invasion and war in Ukraine among PWH with a history of injecting substance use [11]. This study reported the results of 18 interviews with people from four Ukrainian cities in autumn 2022. The study not only outlines personal experiences but also confirms the deep impact of war on the personal and healthcare‐related wellbeing of participants, the necessity to respond to the triggering situations, such as the decrease in work opportunities, incremental costs and the necessity for internal displacement. All these factors were shown to be affecting ART access, adherence and general access to healthcare.

Lazarus et al. analysed the non‐governmental organization (NGO) response during the war and described service provision for key populations in Ukraine, using a mixed‐methods project among geographically dispersed non‐governmental (*n* = 24) and governmental (*n* = 2) organizations representing several Ukrainian regions [12]. Their analysis outlines the impact of the war on NGO work, including HIV prevention and treatment in a time when humanitarian aid is critical. Following the initial shock and the terror of war, the majority of the organizations quickly resumed work, limiting the duration of cessation of services, optimizing response and reallocating work to available staff. This study provides a strong story of resilience and support emphasizing the key work of NGOs in healthcare responses during unfortunate “big events” such as war.

The topic of human rights and the context of war in Ukraine is further expanded by Lohman et al. by presenting an assessment related to human rights barriers to the prevention and treatment of HIV and TB [13]. The study consisted of a series of assessments covering the period of the Russian invasion and outbreak of war aiming to examine the progress in the scale‐up and outcomes of human rights programmes. The paper describes an implementation learning evaluation using 25 interviews with programme implementers, community advocates and government officials, representing 14 organizations. Interview data clearly reflect the reduction of stigma in several domains, including a decrease in the proportion of PWH who faced unauthorized disclosure in the social environment (−19%), a reduction in the number of reported verbal abuses/personal threats (−13%) and improvement (+33%) in the perceived confidentiality of medical records. The war resulted in the expansion of regional coverage of legal and advocacy programmes and services, allowing a large number of the population to maintain access to key services.

Ukraine is also one of few countries in the EECA region that have implemented numerous combination prevention programmes including HIV PrEP, where adherence remains challenging. In the follow‐up of the study on oral daily PrEP with tenofovir disoproxil fumarate (TDF)/emtricitabine (FTC) [14] in 199 people who inject drugs (PWID) in Ukraine with or without the use of text reminders, Morozova et al. analysed longitudinal reported adherence patterns coupled with the measurement of tenofovir diphosphate and emtricitabine triphosphate in dried blood spots from study participants as objective measures of adherence [15]. The study enrolled people who have been injecting drugs for longer than 20 years (37%) with a high proportion of alcohol use (67%) and >50% with moderate to severe depression. In this highly challenging group, the perceived adherence was high to moderate in >90% of the cases with 81% reporting to take more than 95% of their TDF/FTC doses. Alarmingly, data from the dried blood spot metabolite analysis revealed the reality to be divergent from patient‐reported adherence with >50% of participants without detectable TDF/FTC metabolite levels. This study provides unique data confirming the divergence between perceived and measured adherence among PWID, indicating that novel telemedicine or online interventions in combination prevention of HIV can be particularly useful in marginalized or difficult‐to‐reach populations.

Migrants from central Asia moving to central eastern countries to work are disproportionately affected by HIV and sexually transmitted infections due to high‐level stigma and poor working conditions, and have lower or no access to the health system and prevention tools compared to other groups. Kovtun et al. [16] focused on human rights violations related to sexual orientation, gender identity (SOGI), and HIV in six countries in eastern Europe, Caucasus and central Asia in 2022 (Armenia, Kazakhstan, Kyrgyzstan, Tajikistan, Ukraine and Uzbekistan), which have a background of a rapidly rising HIV epidemic, strict social and religious norms, high level of stigma and discrimination associated with sexual and gender minorities and HIV, and regional conflict. Using the Rights—Evidence—ACTion (REAct) tool, they analysed the data on rights violations upon complaints from gay and bisexual men who have sex with men and transgender women. The strikingly higher numbers of rights violations and violations solely based on SOGI in Ukraine compared to the Caucasus and central Asian countries suggest that despite all the achievements made in the country in the last decade in terms of harm reduction, testing, and access to ART and care, stigma and discrimination still prevails in the country. The effect of the recent war in Ukraine was also evident with more human rights violations by police and military staff, denials of private or social services, refusals of temporary accommodation services and denial of border crossings. The results of this study underscore the diversity of the region in terms of stigma, discrimination and human rights violations.

Mackesy‐Amiti et al. define a social intervention that aimed to reduce high‐risk behaviours for HIV among Tajik migrants who inject drugs working in the Russian Federation [17]. This was a cluster‐randomized controlled trial comparing the network‐based, peer educator training with the general health education training. Male Tajik migrants were recruited as peer educators and trained on how to reduce personal risk for HIV acquisition and to deliver knowledge to peers. The interviews showed that the baseline percentages of binge drinking, condomless sex, and syringe and equipment sharing behaviours were considerably high, and needle cleaning behaviour and HIV testing were extremely low in both peer educators and peers. There was a significant reduction in needle‐sharing behaviour following study intervention, but a modest effect on sexual behaviour. This study suggests that interventions tailored to the needs of specific groups and the inclusion of peers may be effective even in populations that are hard to reach.

Stigma‐associated issues were also addressed by Davis et al. who used a citizen science approach to address HIV‐related stigma and increase HIV testing in adolescents and young adults in Kazakhstan [18]. These populations were called to develop digital materials, which would be assessed and rated in a contest with the aim to reduce HIV‐related stigma and promote HIV self‐testing. The submitted materials were judged by a board including peers of the contestants, healthcare professionals and representatives of NGOs, and highly rated submissions were awarded. Adolescents and young adults showed a high level of interest in the project both as contestants, which resulted in a high number of submissions, and as board members providing input in the development and implementation of the study, running social media procedures, creating promotional materials and providing feedback on the submission system. Inclusion of the community stimulated collaboration among adolescents and young adults, and increased knowledge of HIV‐related stigma and the importance of HIV testing.

Lastly, a study also from Kazakhstan on adherence to ART among PWH in association with mental health and cognitive disorders was presented by Mergenova et al. [19]. The size of the HIV epidemic in this country is increasing with the majority of acquisitions reported among people who inject psychoactive substances. The authors performed a cross‐sectional questionnaire‐based analysis of 230 PWH on stable (>6 months) ART, with the assessment of self‐reported ART adherence in addition to depression, anxiety, post‐traumatic stress disorder (PTSD) symptoms, and cognitive and memory assessments. Notably, a third of the patients reported a history of mental illness, while only 25% reported injection drug use and 17% hazardous alcohol drinking. Moreover, mild depression or anxiety was reported in 20−32%, while in 6−10%, these mood disorders were at least moderate including PTSD symptoms observed in 7% of cases. Not surprisingly, these symptoms were associated with lower adherence—missing ART was more likely among participants with mild or moderate depressive symptoms, mild or moderate anxiety symptoms, PTSD symptoms or forgetfulness. On the contrary, better cognitive function was associated with better adherence to therapy. This study emphasizes the need for precise and regular assessment of not only ART but also mental health and cognitive function for better care of PWH in order to tailor specific responses and to provide mental health support for improving treatment adherence and maintaining optimal viral suppression rates.

The long history of neglecting HIV in EECA resulted in rapid increases of transmissions. The high number of PWID and migrants who are disproportionately affected by HIV and the unique characteristics of each country with different cultures, beliefs and structures challenge the long‐term efforts to address the epidemic. This supplement on the HIV epidemic in eastern Europe and central Asia presents a story of resilience, continued efforts to improve human rights, and innovative approaches to combination prevention, and summarizes key challenges related to stigma and other societal issues. While continuing these efforts, further international support, commitment and collaboration will be vital to achieve the new goals for 2030 to end AIDS in the region.

## COMPETING INTERESTS

The authors declare no competing interests.

## AUTHORS’ CONTRIBUTIONS

MP and DG jointly drafted and revised the editorial. All the authors read and approved the final version.

## DISCLAIMER

The authors alone are responsible for the views expressed in this supplement and they do not necessarily represent the views, decisions or policies of the institutions with which they are affiliated.

## Data Availability

Data sharing is not applicable to this article as no datasets were generated or analysed during the current study.
